# Effects of tacrolimus and erythropoietin in experimental spinal cord lesion in rats: functional and histological evaluation

**DOI:** 10.1038/sc.2015.172

**Published:** 2015-10-20

**Authors:** P R de Mesquita Coutinho, A F Cristante, T E P de Barros Filho, R Ferreira, G B dos Santos

**Affiliations:** 1 Spine Surgery Division, Laboratory of Medical Investigation, Instituto de Ortopedia e Traumatologia, Hospital das Clínicas da Faculdade de Medicina da Universidade de São Paulo (IOT-HCFMUSP), São Paulo, Brazil; 2 Spine Surgery Division, Laboratory of Medical Investigation, Spine Surgery Division, IOT-HCFMUSP, São Paulo, Brazil; 3 Laboratory of Medical Investigation, Spine Surgery Division, IOT-HCFMUSP, São Paulo, Brazil; 4 Spine Surgery Division, Laboratory of Medical Investigation, IOT-HCFMUSP, São Paulo, Brazil; 5 Laboratory of Medical Investigation – 41 (LIM-41), IOT-HCFMUSP, São Paulo, Brazil; 6 Instituto de Ortopedia, Faculdade de Medicina, Universidade de São Paulo, São Paulo, Brazil

## Abstract

**Study design:**

Experimental study with rats.

**Objective:**

To evaluate functional and histological effects of tacrolimus (FK 506) and erythropoietin (EPO) after experimental spinal cord contusion injury (SCI).

**Setting:**

Brazil.

**Methods:**

Wistar rats (*n*=60) were submitted to SCI with the NYU Impactor system. The control group received saline; the EPO group received EPO; the group EPO+FK 506 received EPO associated with tacrolimus and the group FK 506 received tacrolimus only. The Sham group underwent SCI, but did not receive any drug. Locomotor function was evaluated after SCI by BBB (Basso, Beattie and Bresnahan) weekly and by the motor-evoked potential test in 42 days. The spinal cord was histologically evaluated.

**Results:**

There was a significant difference between treated and the control groups from the seventh day on for BBB scores, with no difference between the groups EPO and EPO+FK 506 by the end of the study. There were significant differences between groups for necrosis and bleeding, but not for hiperemia, degeneration and cellular infiltrate. Axon neuron count was different between all groups (*P*=0.001), between EPO+FK 506 and FK 506 (*P*=0.011) and between EPO+FK 506 and Sham (*P*=0.002). Amplitude was significantly different between all groups except between control and sham. For latency, there was no difference.

**Conclusions:**

This study did not reveal significant differences in the recovery of locomotor function, or in the histological and electrophysiological analysis in animals treated with EPO and tacrolimus after thoracic SCI.

## Introduction

In the last decade, research efforts have concentrated in reducing the secondary lesion after spinal cord lesion, in order to promote axonal regeneration. However, there is no pharmacological treatment with proven benefit yet.^1^ There are weak evidences of some effect of metilprednisolone in high doses, administered shortly after the lesion,^2, 3, 4^ and although there are respiratory complications, sepsis and gastrointestinal bleeding,^1^ the drug is still used in spinal trauma in many institutions.^5, 6, 7^ There are side effects of this therapy, which impair neuronal regeneration, by inhibiting the activity of immune cells, processing and elimination of antigens by macrophages, causing mild neutropenia, exacerbation of post-ischemic necrosis and inhibition of axonal sprouting.^7^ Other drugs have been investigated, such as estrogen,^8^ agonists of estrogen receptors,^9^ progesterone,^10^ erythropoietin (EPO),^11^ magnesium^1^ and immunophilins binders, such as tacrolimus (FK 506).^12^

EPO is a hematopoietic growth factor produced in the kidney of adults and in the liver of fetuses, which stimulates the proliferation and differentiation of erythroid progenitor cells. Many mechanisms, acting in different phases of the secondary damage cascade after SCI,^11, 13^ are implicated in the neuroprotective effect of EPO. They are referred to in the literature as protectors against the central nervous system lesion after ischemia,^14^ they have antiapoptotic^15^ and anti-inflammatory roles,^16^ and they are lipid peroxidation inhibitors.^17^ It was demonstrated by immunohistochemistry that EPO is able to cross the blood–brain barrier and bind to receptors, abundant in the central nervous system, in capillaries within the white matter, in the body and proximal dendrites of motor neurons and in the anterior horn of the spinal cord.^14^ A marked neuroprotective effect of EPO was observed after ischemic injury and reperfusion, which can take place after cardiological procedures, in which there is diminished blood supply to the spinal cord, as the expression of EPO and its receptor is modulated by hypoxemia.^14^

Immunophilins belong to a family of proteins that are receptors for immunosuppressive drugs, such as cyclosporin A, FK 506 (tacrolimus) and their non-immunosuppressive analogs thereof, which are collectively called immunophilin ligands.^12^ Their concentration in the central nervous system is 50 times higher than in immune system cells.^12, 18^ The word ‘tacrolimus' is based on *Tsukuba macrolide immunosupressant,* a macrolide antibiotic produced by the bacterium *Streptomyces tsukubaensis*, discovered and isolated in 1984 from a soil sample obtained in Japan.^19^ Tacrolimus is an immunosuppressant drug, which is able to cross the blood–brain barrier, and used to prevent rejection in allogeneic transplants.

There are different types of receptors for tacrolimus that can mediate the immunosupression or the neuronal regeneration.^20^ The FK*-binding protein* (FKBP-12) is a cytoplasmic protein, with molecular weight of 12 kDa, and which is responsible for the immunosuppressive activity of tacrolimus via calcineurin inhibition.^12, 20^ The neurotrophic effect occurs by a mechanism independent of the activation of calcineurin, after binding to the receptor protein of 52 kDa: FKBP-52.^18, 20, 21^ It increases the expression of the neuronal protein 43, associated with the growth (CAP-43) in sensory neurons in the dorsal root ganglia in rats.^22, 23^ FK 506 has also other functions: restoration of depleted reserves of mitochondrial ATP, reduction of mitochondrial swelling and cellular oxidation index, promotion of the elevation of glutathione^24^ and protection of axons against secondary injury after spinal cord lesion.^25^ In experimental models, FK 506 improved nerve regeneration in traumatic neural injuries^26^ and in neurodegenerative diseases.^27^

## Objective

The objective of this study was to investigate the effects of tacrolimus and EPO as neuroprotective and neurotrophic agents, isolated or in association, in experimental SCI. The hypothesis was that the two drugs would have a synergistic effect on functional and histological recovery from the SCI.

## Materials and methods

### Study design, ethics and animals

In this experimental study with rats, all institutional and governmental regulations, and all international guidelines regarding the care of experimental animals and pain control, were followed. The research protocol was approved by the Research Ethics Committee of the Institution.

The experimental lesions, pharmacological therapy and functional and histological analyses were performed in the same university laboratory. Five animals were housed in each cage, and they were handled and stimulated to move before the experiment, so that they could become accustomed to the researchers and to the experiment motor function evaluation after the SCI. Rats were maintained in 60 × 40 cm^2^ cages (up to five per cage), in a controlled temperature room (25 °C) and with *ad libitum* feeding and hydration for the whole duration of the study.

We used Wistar, male, adult rats, weighing between 320 and 340 g. They were all healthy, presented normal fur and normal motricity, and were evaluated and followed up by the laboratory veterinarian.

We excluded the rat from the study in the following cases:

Death following the spinal cord lesion;

Skin changes in the incision area;

Autophagia or mutilation behavior;

Deep infection, refractory to antibiotic therapy;

Urinary infection even after 10 days of antibiotic therapy (blood in the urine);

Normal movements after the lesion (21 points in the BBB (Basso, Beattie and Bresnahan) scale);

BBB score of 3 points or higher in the lower limbs after the lesion.

All rats were submitted to a thoracic SCI with the NYU Weight-Drop Impactor (John A Gruner, Department of Neurosurgery, New York University Medical Center, New York, NY, USA), and were evaluated by the BBB scale and by the motor-evoked potential (MEP) exam. After euthanasia, tissues from the spinal cord were evaluated histologically as detailed below.

### Sample and allocation

The sample was calculated using the formula *N*=(SD/*d*)^2^, where SD stands for standard deviation and *d* is the value of Cohen.^28^ By using 80% power, and an SD of 2.8 and a *d* value of 0.8, the sample obtained should be of 12 animals per group. We used 60 Wistar rats divided into five groups, which received the interventions described in Table 1.

### Surgical procedures and spinal cord lesion

Before laminectomy, experimental cord lesion, electrophisiological exam and euthanasia, the animals were anesthetized with 50 mg kg^−1^ of ketamine and 10 mg kg^−1^ of xylazine.^29, 30, 31^ The rats were examined to confirm anesthesia.^29^

Rats were submitted to laminectomy from T8 to T12, with hemostasis and suture with nylon monofilament (3.0) as described before.^29, 31, 32^ The NYU Impactor equipment was used to produce a moderate spinal cord lesion,^32^ with the drop of a weight of 10 g from a 25-mm height.

Animals received intraperitoneal antibiotic (cefazolin sodium, 5 mg kg^−1^) immediately after injury and once daily for the next 3 days. After surgery, all rats were given 2 mg kg^−1^ of meloxicam once daily for 7 days, and 5 mg per 100 g of tramadol hydrochloride intramuscularly for 5 days. The animals' bladders were manually emptied between 6 and 24 h after the lesion and then daily until they were killed.

### Evaluation of locomotor function: BBB and MEP tests

Recovery of locomotor function following the spinal cord lesion was evaluated using the BBB scale on days 2, 7, 14, 21, 28, 35 and 42 after the spinal cord lesion, following the protocol routinely used by our laboratory.^29, 30, 33^ The assessment of each rat was conducted simultaneously by two suitably trained observers, blinded to group allocation and to their colleague's evaluations. When there was disagreement between the evaluations, the lower score was recorded for analysis.

**Figure 1:**
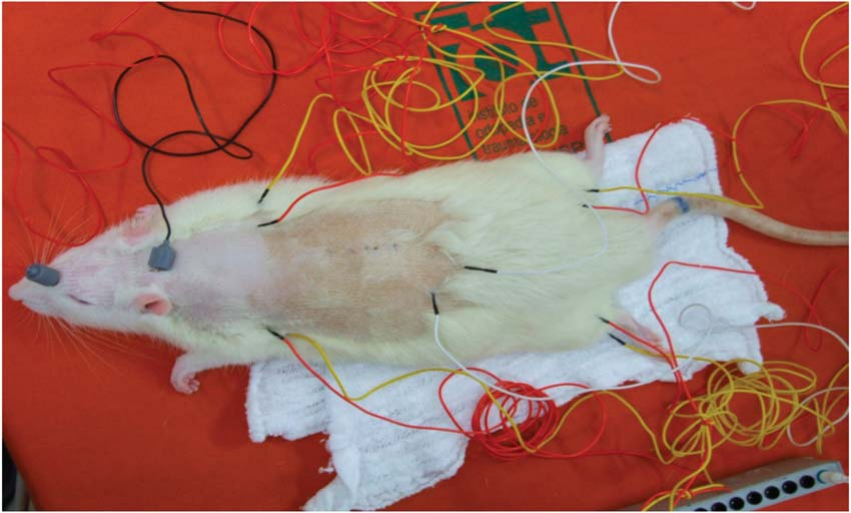
Positioning of electrodes on the anesthetized rat for the motor-evoked potential exam.

Neurological deficit after spinal cord lesion was evaluated by the MEP exam in the 42th day,^34^ using a four-channel electromyography machine, with monopolar needle electrodes of the corkscrew type (E0401, Neuromedical). A single intraperitoneal dose of pentobarbital was administered (55–75 mg kg^−1^), and ketamine (also 55–75 mg kg^−1^) was given intramuscularly. After shaving, one monopolar needle electrode was placed in the lumbar region as a ground and two corkscrew-type electrode needles in the head of the rat, on the inter-hemispheric line in the frontal (anode) and occipital (cathode) regions for simultaneous bilateral stimulation. The capture of muscular responses was performed by inserting pairs of monopolar needle electrodes, with defined and fixed inter-electrode distance, into the proximal muscles of fore and hind limbs, as shown in Figure 1.

For instrument calibration, the following parameters were used in the capture of muscular responses: 20 ms window, sensitivity 2 mV/div, low-frequency filter (10 Hz) and high frequency of 10 kHz, and transcranial electrical stimulation through single stimulus of 0.2 ms.

### Histological study

After MEP, 42 days after injury, the animals were killed and their spinal cords were examined histologically. For euthanasia, an injection of 140 mg kg^−1^ of intraperitoneal pentobarbital was administered after anesthesia. The spinal cord was dissected from C3 to T10 and fixed in formalin at 10%.

The tissues were sectioned and the fragments were bathed in alcohol, diaphanized in xylol and embedded in paraffin Paraffin blocks were cut in up to 5-μm-thick sections (Leica microtome MR 2055, Leica, Wetzlar, Germany) and the material was disposed in glass slides, previously bathed in saline. The slides were stained with hematoxylin and eosin.

One single pathologist, blind to animal allocation, made all histological analyses. He evaluated the following histopathological variables with a score varying from 0 (absent) to 3 (intense): necrosis, hemorrhage, hyperemia, nerve degeneration and cellular infiltrate.

**Figure 2:**
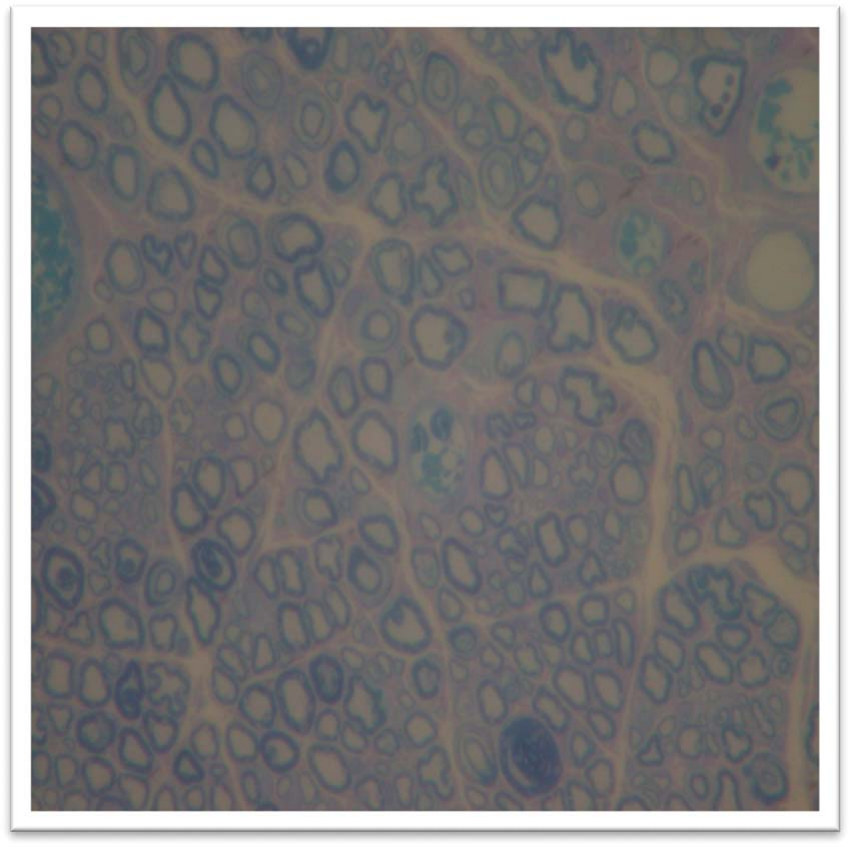
Blue toluidine staining of the spinal cord for axon neural count (× 40).

In addition, the pathology performed axon neuron count. For this, the spinal cord sections were fixed in osmium tetroxide solution and stained with 2% toluidine blue, at 1%, and two areas with good representation of cells were chosen from each section. Images were analyzed at × 40 magnification (example is given in Figure 2) using the Sigma Scan Pro5.0 software (Sigma, San Jose, CA, USA) for regenerated axons fibers counting. Only neurons with diameters greater than 15 μm were considered for counting.

### Statistical analysis

Data were registered in spreadsheets and analyzed using the SPSS 20.0 for Mac software (Armonk, NY, USA). The primary outcome was the BBB score in the 42th day. Secondary outcomes were the histological and MEP findings. Normality was tested. Mann–Whitney test was used in pairwise comparisons between two groups and Kruskal–Wallis for the analysis between all groups. One-way analysis of variance was used in BBB scores, cell count and variables of histological analysis. Analysis of variance for repeated measures was used for follow-up. Type I error *P*⩽0.05 was accepted.

## Results

During the study, two rats in each group died. In the control group, rats #2 and 6 died of infection, in the first and third weeks. In the EPO group, rat #5 died for autophagia in the fourth week and rat #10 died of unknown cause at the second week. In the EPO+FK 506 group, rat #2 died in the third week of autophagia and rat# 4 was excluded due to a resistant urinary infection. In the FK 506 group, rat #6 was excluded due to autophagia and rat #9 died of infection in the second week. In the Sham group, rat #4 died, in the second week, of urinary infection, and rat #1 was excluded due to autophagia.

### Functional analysis

**Figure 3:**
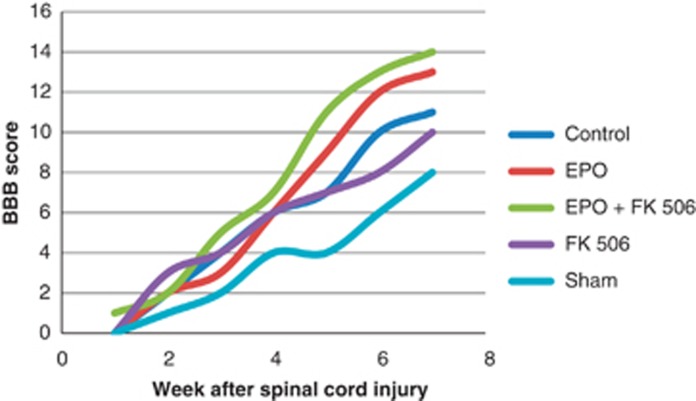
Evolution of the BBB scores in the study moments according to groups.

From the seventh day after SCI, Kruskal–Wallis test and analysis of variance have shown that the BBB scores were significantly different between groups, as shown in Table 2, but there was a progressive increase in BBB scores in all groups, as shown in Figure 3. Pairwise *post hoc* comparisons were made weekly, and in the seventh week, EPO and EPO+FK 506 groups were significantly different from the Control group (Table 3). Nevertheless, in the end of the study, the difference was not significant anymore.

### Histological analysis

Table 4 shows the scores for histological analysis for all variables. For necrosis, significant differences were found in pairwise comparisons between Control and EPO+FK 506 (*P*=0.009), EPO and FK 506 (*P*=0.023) and EPO+FK 506 and FK 506 (0.004). Control and Sham groups were similar for necrosis (*P*=0.054). For bleeding, EPO versus FK 506 were significantly different (*P*=0.014), as were EPO+FK 506 versus FK 506 (*P*=0.001) and FK 506 versus Sham (*P*=0.035). However, hyperemia, degeneration and cell infiltrate scores were similar.

Axon neuron count analysis by analysis of variance has shown significant differences between all groups (*P*=0.001). Significant differences were also found in pairwise comparisons between EPO+FK 506 and FK 506 (*P*=0.011) and between EPO+FK 506 and Sham (*P*=0.002).

### MEP exam

There were significant differences in MEP results for amplitude for all groups (*P*<0.05), except for the comparison between the Control and the Sham groups. As shown in Table 5, all pairs were significantly different except for Sham versus EPO+FK 506 and Sham versus EPO.

## Discussion

In this study, a significant improvement of locomotor function was observed in all rats from the 2nd to the 42nd day after SCI. The BBB scores showed paralysis (score 0) between the second and seventh days after SCI, which suggests there is a transient interruption of the spinal cord physiological function that is apparently more severe than the neurological deficit, which will be definitive. This is in line with the postulate by Basso *et al.*^35^ Although the primary mechanical injury is usually irreversible, there is a cascade of biological events that result in the secondary SCI, which can be ameliorated by neuroprotective drugs.^36^ We chose to study EPO and tacrolimus because, according to several authors, these substances have resulted in improved neurological function.^14, 15, 16, 17^ Many mechanisms are proposed to explain the neuroprotective effects of EPO against post-ischemic injury of the central nervous system:^14^ they are accepted as modulators of the antiapoptotic function,^15^ with anti-inflammatory function^16^ as well as lipid peroxidation inhibitors.^17^ The effects of tacrolimus are mentioned in the literature as restoring the depleted reserves of mitochondrial ATP, reducing mitochondrial edema and cellular oxidation index, promoting the elevation of glutathione^24^ and protecting axons from the secondary injury after SCI.^25^

From the third week, the functional recovery of BBB scores was faster in the EPO compared with the Control group, although not statistically significant, but in the sixth week of the study, the animals of the EPO group had significantly higher BBB scores. The faster progression and the higher BBB score in the EPO group suggest a possible mitigating effect on secondary SCI. EPO proved its therapeutical effect, as shown by other authors.^37^ The delay in the results (with statistical significance only in the sixth week of the study) may be attributed to differences between studies in experimental design, animal race, sex and weight, the type of device employed to induce SCI, and different biomechanical characteristics of the lesion. The drug was also administered differently between studies regarding timing and frequency. Although our results do not invalidate the potential neuroprotective effect of EPO previously documented by other researchers,^11, 16^ they point to the need for further experimental research to refine therapeutic approach in SCI.

The same occurred with EPO+FK 506: only in the sixth week the combination of drugs showed superior results in the BBB score than in the Control group. Besides, the combination was not significantly different from the use of isolated EPO but it was different from tacrolimus. This suggests that there is no synergistic effect of the two drugs regarding the functional evaluation. Besides, FK 506 was not superior than the Control group, and BBB scores with tacrolimus were lower than with EPO and than with the combination of the drugs. Therefore, it seems that EPO, and not tacrolimus, is the responsible for the higher BBB scores. The literature has shown similar^38^ and different^39, 40^ results, possibly due to different methodology in the investigations.

Also in the histological analysis, the synergistic effect of EPO and tacrolimus failed to show. There was no reduction of the severe cases of necrosis and no reduction in the higher scores of bleeding. For axon neuron count, significant differences were found in pairwise comparisons between EPO+FK 506 and FK 506 and between EPO+FK 506 and Sham. However, there is no other study, to the best of our knowledge, addressing EPO and tacrolimus in combination, so that the true meaning of this finding is yet to be further investigated.

In MEP, amplitude indicates recovery or gain of motor fibers and latency indicates the time taken for the electrical sign to show up, that is, it represents the conduction of the electrical impulse through the axon pathways that were not damaged in the process of SCI. In our study, unexpectedly, tacrolimus, either isolated or in combination with EPO, resulted in a reduction in the mean amplitude and increase in latency, therefore a worse result. We did not find any correlation between MEP and histological results, unlike Basso *et al.*,^41^ who noticed a direct correlation between the remaining viable neural tissue and functional recovery. However, that study used electron microscopy scanning, with a special technique for neural tissue, whereas in our study we used light microscopy to evaluate the pathologic findings after SCI.

Therefore, although both EPO and tacrolimus have shown neuroprotective results after SCI in other studies, we were not able to identify, in our investigation, the exact timing and dosage of a combination treatment that would result in clinical effects.

Therefore, although both EPO and tacrolimus have shown neuroprotective results after SCI in other studies, we were not able to identify, in our investigation, the exact timing and dosage of a combination treatment that would result in clinical effects. Both tacrolimus and EPO are under study for their neuroprotective effects, but the ideal dosage has not yet been established, neither the window of time for the neuroprotective effects. The possible benefits of the drugs are not discarded, and neither are the synergistic effects, and other studies, investigating different dosages, are warranted. Although our results do not discard the neuroprotective effects found before,^11, 16, 42^ they point out to the need of more detailed experimental investigations, with the control for confounding factors such as race, sex and weight of the animals, the biomechanical conditions of experimental spinal cord lesions and timing and dosage of drug administration.

## Conclusion

Our experimental study was not able to find differences in the recovery of locomotor function, the histological or electrophysiological exams between rats undergoing treatment with EPO, tacrolimus or both after thoracic spinal cord contusion injury.

## Data Archiving

There were no data to deposit.

## Figures and Tables

**Table 1:** Interventions in each study group

*Group*	n	*Summary of interventions*
Control	12	SCI; 1 ml of saline (0.9%) at 5 min
EPO	12	SCI; EPO (1000 UI kg^−1^) at 5 min
EPO+FK 506	12	SCI; EPO (1000 UI kg^−1^)+tacrolimus (1 mg kg^−1^) at 5 min
FK 506	12	SCI; tacrolimus (1 mg kg^−1^) at 5 min
Sham	12	SCI; no drug administration

Abbreviations: EPO, erythropoietin; SCI, spinal cord injury.

**Table 2:** Results of functional evaluation using the BBB scale according to groups and moment of evaluation after spinal cord lesion

*Group*	*Rat*	*Day 2*	*Day 7*	*Day 14*	*Day 21*	*Day 28*	*Day 35*	*Day 42*
Control	1	0	2	4	6	8	12	14
	**2**	0	Death	—	—	—	—	—
	3	0	0	5	6	9	13	14
	4	0	1	5	4	5	9	14
	5	0	2	3	3	4	8	8
	**6**	0	0	2	Death	—	—	—
	7	1	1	3	4	5	5	6
	8	0	1	1	3	2	4	5
	9	0	4	6	8	9	13	13
	10	0	3	3	7	12	13	12
	11	0	3	5	7	9	14	12
	12	0	2	4	7	8	9	12
EPO	1	0	4	6	7	9	14	16
	2	0	4	5	7	12	15	16
	3	0	1	1	2	8	13	13
	4	1	2	4	5	4	12	12
	**5**	0	0	1	0	Death	—	—
	6	0	2	2	2	6	9	10
	7	0	2	4	7	8	12	15
	8	0	0	2	5	12	14	14
	9	1	3	4	9	13	14	14
	**10**	1	0	Death	—	—	—	—
	11	1	3	4	6	8	6	11
	12	0	1	2	5	7	9	12
EPO+FK 506	1	0	0	3	8	14	15	16
	**2**	1	0	1	Death	—	—	—
	3	1	6	8	9	13	17	17
	**4**	—	—	—	—	—	—	—
	5	1	5	6	12	12	14	15
	6	1	0	2	4	8	7	12
	7	0	4	7	8	9	12	15
	8	0	0	6	7	9	14	13
	9	1	0	5	7	12	14	17
	10	0	0	6	4	8	12	13
	11	0	2	4	7	12	11	11
	12	1	0	3	8	12	11	11
FK 506	1	0	2	3	7	4	6	6
	2	0	4	6	8	11	12	13
	3	0	5	6	7	5	8	9
	4	1	4	6	8	8	5	7
	5	0	4	5	8	12	14	16
	**6**	—	—	—	—	—	—	—
	7	0	4	3	2	7	8	11
	8	0	3	4	6	8	6	6
	**9**	0	0	Death	—	—	—	—
	10	0	0	2	4	3	12	15
	11	0	2	2	3	5	4	4
	12	2	5	6	4	6	9	11
Sham	**1**	—	—	—	—	—	—	—
	2	1	1	1	3	5	5	7
	3	0	0	3	3	9	12	12
	**4**	0	0	Death	—	—	—	—
	5	0	0	4	3	4	8	8
	6	0	1	2	2	3	5	8
	7	0	0	0	0	0	1	4
	8	1	3	3	4	5	5	8
	9	0	1	4	8	5	6	6
	10	1	1	2	6	4	5	8
	11	0	0	0	7	6	8	10
	12	0	3	4	3	2	4	7

Abbreviations: BBB, Basso, Beattie and Bresnahan; EPO, erythropoietin. The number in bold are those of animals that died or were excluded.

**Table 3:** *P*-values for the comparison of BBB scores between groups (Mann–Whitney test) according to the study week

*Groups*	*W1*	*W2*	*W3*	*W4*	*W5*	*W6*
Control versus EPO	0.605	0.495	0.482	0.167	0.83	0.042
Control versus EPO+FK 506	0.205	0.095	0.028	0.005	0.80	0.032
Control versus FK 506	0.047	0.143	0.701	0.195	0.032	0.109
Control versus Sham	0.007	0.049	0.094	0.004	0.002	0.004
EPO versus EPO+FK 506	0.528	0.032	0.014	0.115	0.889	0.929
EPO versus FK 506	0.035	0.045	0.731	0.011	0.000	0.001
EPO versus Sham	0.062	0.260	0.400	0.000	0.000	0.000
EPO+FK 506 versus FK 506	0.030	0.410	0.019	0.000	0.000	0.001
EPO+FK 506 versus Sham	0,316	0.007	0.001	0.000	0.000	0.000
FK 506 versus Sham	0.000	0.008	0.272	0.097	0.224	0.465

Abbreviations: BBB, Basso, Beattie and Bresnahan; EPO, erythropoietin; W, week.

**Table 4:** Histological evaluation scores for necrosis, bleeding, hyperemia, degeneration and cellular infiltrate

*Group*	*Absent*	*Mild*	*Moderate*	*Intense*	*Total*
*Necrosis*					
Control	3	2	4	0	9
EPO	1	7	1	0	9
EPO+FK 506	1	9	0	0	10
FK 506	0	2	4	4	10
Sham	0	6	2	2	10

*Bleeding*					
Control	1	5	3	0	9
EPO	1	6	2	0	9
EPO+FK 506	1	8	0	1	10
FK 506	1	0	8	1	10
Sham	0	5	3	2	10

*Hyperemia*					
Control	2	5	1	1	9
EPO	0	7	2	0	9
EPO+FK 506	2	5	2	1	10
FK 506	0	4	5	1	10
Sham	0	2	5	3	10

*Degeneration*					
Control	3	4	2	0	9
EPO	3	2	4	0	9
EPO+FK 506	2	4	4	0	10
FK 506	0	3	3	4	10
Sham	0	4	3	3	10

*Cellular infiltrate*					
Control	1	4	3	1	9
EPO	2	3	3	1	9
EPO+FK 506	1	3	4	2	10
FK 506	0	3	3	4	10
Sham	0	5	5	0	10

Abbreviation: EPO, erythropoietin.

**Table 5:** *P*-values for the comparison between groups for mean amplitude and latency, according to the MEP exam

*Groups*	*Amplitude (mA)*	*Latency (ms)*
Control versus EPO	0.045	0.001
Control versus EPO+FK 506	0.000	0.000
Control versus FK 506	0.000	0.000
Control versus Sham	0.903	0.000
EPO versus EPO+FK 506	0.000	0.002
EPO versus FK 506	0.000	0.000
EPO versus Sham	0.024	0.151
EPO+FK 506 versus FK 506	0.001	0.001
EPO+FK 506 versus Sham	0.001	0.093
FK 506 versus Sham	0.000	0.000

Abbreviations: EPO, erythropoietin; MEP, motor-evoked potential.
